# Peer support and social networking interventions in diabetes self-management in Kenya and Uganda: A scoping review

**DOI:** 10.1371/journal.pone.0273722

**Published:** 2022-09-26

**Authors:** Habil Otanga, Brian Semujju, Lynn Mwaniki, Justus Aungo

**Affiliations:** 1 Department of Psychology, University of Nairobi, Mombasa Campus, Mombasa, Kenya; 2 Department of Journalism and Communication, Makerere University, Kampala, Uganda; 3 Department of Sociology, University of Nairobi, Mombasa Campus, Mombasa, Kenya; Flinders University, AUSTRALIA

## Abstract

**Background:**

Diabetes mellitus is a growing worldwide health challenge especially in sub-Saharan Africa. While the use and effectiveness of diabetes self-management interventions is well documented in high-income countries, little information exists in sub-Saharan Africa. Therefore, this study attempted to synthesize information in the literature on the use and efficacy of peer support and social networking in diabetes self-management in Kenya and Uganda.

**Objective:**

The purpose of this scoping review is to summarize research on the extent of use and efficacy of peer support and social networking interventions in diabetes self-management in Kenya and Uganda.

**Design:**

We searched PubMed, ScienceDirect and Cochrane Library databases for articles reporting peer support and social networking interventions in Kenya and Uganda published in English between 2000 and September 2021. Key words encapsulated three major themes: peer support, social networking and self-management. Hand searches were also conducted to select eligible papers. Data was extracted using a form prepared and piloted in line with PRISMA-ScR guidelines.

**Results:**

Thirteen peer reviewed articles were selected for analysis. Eleven studies reported peer support interventions while two focused on social networks in diabetes self-management. Peer support and social networking interventions incorporated microfinance and group medical visits, diabetes self-management education, telephone support and Medication Adherence Clubs. Most interventions were delivered by multidisciplinary teams comprising nurses and other professionals, peer educators, peer leaders and community health workers. Most interventions were effective and led to improvements in HbA1c and blood pressure, eating behaviors and physical activity and social support.

**Conclusions:**

The limited studies available show that peer support and social networking interventions have mixed results on health and other outcomes. Importantly, most studies reported significant improvements in clinical outcomes. Further research is needed on the nature and mechanisms through which peer support and social network characteristics affect health outcomes.

## Introduction

Diabetes is a growing worldwide health challenge especially in sub-Saharan Africa. By the end of 2019, an estimated 19.4 million adults aged 20–79 years were living with diabetes in the International Diabetes Federation (IDF) Africa region, representing a regional prevalence of 3.9% [1]. In Uganda, an estimated 300,000 of the 19 million adults in Uganda live with diabetes [2]. Diabetes prevalence in Kenya is estimated at 3.3% and is projected to rise to 4.5% by 2025. The strain of diabetes on healthcare systems in resource-poor countries [3] that suffer from inadequately trained healthcare workers and lack of affordable healthcare is an issue of concern [4]. To meet this challenge, self-management has increasingly become a preferred option for patients. However, few diabetes support interventions explore means of improving self-management within social support networks, yet for people with long-term conditions, social networks can provide an important means of mobilizing, mediating and accessing support [5]. Peer and social support interventions emphasize mutuality and psychosocial interactive connections and social capital accumulation to enhance patient confidence and capacity for compliance and medication adherence and use of new technologies [6]. Linkages between social relationships and health are achieved through the control of health habits and social control. For instance, family members and friends facilitate self-management through assisting patients to follow aspects of self-care [7, 8] through social and practical support [9, 10] and encouragement and reminders of medication among others. In sub-Saharan Africa (SSA), such social networks play an increasingly important role in health behavior in the absence of formal support systems by providing perceived or actual social support, social influence and access to resources and provide avenues for the spread of attitudes, behaviors and financial, informational and social resources [11, 12].

There are wide variations of content and structure, roles and interactions, and settings and activities of peer support, ranging from structured and unstructured models [13]. However, all peer support relationships are anchored in a non-hierarchical nature and reciprocity [14]. In SSA, diabetes self-management education (DSME) is provided mainly in primary health care (PHC) settings but also in community settings. Both healthcare and non-healthcare professionals provide DSME and social support [15]. For instance, reviews [16, 17] report the use of medical professionals-led [18–21] and non-medical professionals-led support programs and those employing multidisciplinary teams [22–24]. Most interventions employ group meetings in tandem with home visits and mobile phone support [25–27]. The duration of interventions ranges from 5 weeks to 48 months and 1–10 days to train peer supporters to deliver the interventions [28, 29]. A recent peer support model in diabetes self-management is the combination of microfinance and group medical visits that operate synergistically to enhance health and economic outcomes [30]. Such synergy builds social capital, self-reliance and address social disadvantage [31] and responds to economic barriers to healthcare access wrought by cutbacks in healthcare financing in resource-limited countries [32, 33].

DSME interventions are associated with improvements in self-care behaviors (diet, foot care, physical activity), diabetes knowledge, coping and stress relief [34–36]; significant clinical outcomes (glycemic control) [24]; and acceptability [37]. However, these interventions suffer low attendance and high attrition [37–39] due to cost, access and poor diabetes knowledge. On their part, telephone-based interventions suffer poor attendance and reach [40], non-significant improvements in clinical outcomes, dietary adherence and physical activity [26, 41]. Contrastingly, they are associated with positive outcomes including increase in sleep, social support coping and retention rates [26].

Whereas the use and efficacy of peer support interventions has been established in high income countries (HIC), limited data restricts drawing similar conclusions in SSA. It remains unclear what kind of peer support and social networking interventions are used in Kenya and Uganda. Therefore, a scoping study was conducted to systematically map literature in this area and identify gaps in research and practice. The following research question was formulated: What is known from existing literature about the use of peer support and social networking interventions in diabetes self-management in Kenya and Uganda?

## Materials and methods

Ethical approval was provided by the Kenya Medical Research Institute (KEMRI) Protocol No. 4295. Research approval was provided by the National Commission for Science, Technology and Innovation (NACOSTI) Permit number: NACOSTI/P/21/13911.

### Study design

We employed a scoping design to carry out an overview of research evidence on the use of peer support and social networking interventions for diabetes management in Kenya and Uganda. These interventions are relatively new in sub-Saharan Africa, and to the best of our knowledge, this study is the first attempt to systematically examine the breadth of research on the use of peer support and social networking interventions in the East African region. With little research evidence available and negligible randomized controlled trials done, it is difficult to undertake a systematic review, hence making this study exploratory [42, 43]. Consequently, we set out to include all studies [42] with no attempt made to appraise the quality of the evidence or carry out an assessment of risk of bias of studies. We included a greater range of study designs and methodologies [43] to provide an overview of where, how and what peer support and social networking interventions are available in diabetes management in Kenya and Uganda. Therefore, this design helped us to compile, categorize and describe available peer support and social networking models of diabetes management and their outcomes and identify gaps in the evidence base [44]. By so doing, the scoping review helped to contextualize knowledge by identifying what we know and do not know about peer support and social networking interventions in diabetes management in the two countries. This study was conducted using a framework for scoping reviews [44] and the Preferred Reporting Items for Systematic Reviews and Meta-Analyses extension for Scoping Reviews (PRISMA-ScR) checklist and flow diagram [45]. The protocol for this scoping study was not registered but can be accessed on request from the corresponding author.

### Search strategy

We searched PubMed, Sciencedirect and Cochrane Library using key terms and Boolean operators related to 1) diabetes/diabetes mellitus; 2) peer support/social support/peer group*/social group*/peer support group*; 3) peer network*/social network*/support network*/social support network*. The search strategies were drafted by a Librarian from Makerere University and refined by the research team. The final search strategy for PubMed can be found in S1 Appendix. The search was completed in September 2021 and limited to peer reviewed papers reporting interventions in Kenya and Uganda published in English between 2000 and 2021. Database searches were supplemented by hand-searching reference lists of recent review articles.

### Study selection

Two researchers independently screened titles and abstracts for relevance based on the inclusion and exclusion criteria established a priori and refined in a pilot using the first 20 studies identified. Records were then consolidated in an Excel sheet where duplicates were removed. Two researchers verified the screening for accuracy. Any disagreements on study selection and data extraction were resolved by discussion. Citations were excluded if they did not appear to be relevant to the topic of peer support and social networking in diabetes management. Following the initial title/abstract screening and removal of duplicates, two investigators screened the full texts and articles that met the inclusion criteria qualified for data extraction while those that failed to meet the criteria were tagged indicating the reason for exclusion.

The following inclusion criteria were used in study selection: 1) published peer reviewed papers describing peer support and/or social networking interventions in diabetes self-management; 2) examined peer support and/or social networking intervention outcomes on diabetes self-management; 3) available in English; 4) interventions described were carried out in Kenya or Uganda only. Articles were also included if the peer/social networking intervention targeted more than a single non communicable disease (NCD) in one setting. Qualitative, quantitative and mixed methods articles were included to capture characteristics of peer support and social networking interventions, outcomes, efficacy and challenges. Articles were excluded if they were commentaries, conference presentations, reviews, protocols, studies describing an intervention carried out in multiple countries including Kenya or Uganda, studies whose full-text article was unavailable, and studies that reported PHC-based interventions without any peer dimension. The most recent reviews were used for reference list search.

### Data charting

One researcher used a detailed charting form to extract data from each eligible article. One researcher verified the data charted and any disagreements were resolved by consensus of the four researchers. The form captured the following key information: article characteristics (author, year), study characteristics (sample size, study design, location) and intervention characteristics (name of the intervention, who delivered the intervention, how intervention was delivered, duration and outcomes of interventions).

### Collating, summarizing and reporting results

To present an overview of all material reviewed a narrative account of existing literature was preferred in line with the recommendations of Arksey & O’Malley. First, a numerical analysis of the nature and distribution of interventions, populations and outcomes was summarized in a table (Table 1) under the headings; year of publication, country where the study was conducted, demographic profile of participants, study design, mode of delivery of peer support and social networking and key outcomes. This informed the main areas of focus and gaps in diabetes self-management in the two countries. Secondly, literature was then organized thematically [43] and presented descriptively according to intervention characteristics, outcomes and efficacy of interventions.

**Table 1:** Characteristics of included studies.

Author/Year	Country of study & setting	Demographic profile of participants	Study design	Mode of delivery of PS/SN	Key outcomes/Findings
Baumann et al., 2015 [46]	Uganda Rural	46 adults with diabetes	Pre-post quasi-experimental design	Short-term telephone- based peer support program compared with routine care with no phone-based support -pairing peers and partners	Change in diabetes self-care activities and glycemic control Change in social support and emotional well-being Linkage to care Sustainability of intervention
Khabala et al., 2015 [47]	Kenya Urban	1432 HIV, hypertension and diabetes patients	Retrospective, descriptive study	Medication Adherence Clubs for care of HIV, Hypertension and DM compared to routine care without MACs	Feasibility and early efficacy of MACs on care Increased adherence to treatment
Pastakia et al., 2013 [48]	Kenya Peri-urban and rural	582 adults including 346 people with diabetes	Cohort study design	Community-based screening for DM and hypertension compared to home-based screening	Screening, referral and follow-up within 3 months Feasibility of community- and home-based screening for HTN and T2DM Low rates of follow up
Mwangi et al., 2020 [49]	Kenya Rural	N = 734: Intervention arm: 369 participants + 14 peer educators; Control arm: 365 participants 7 clusters each	Mixed methods process evaluation of a cluster RCT	Comparison of effectiveness of a peer-led health education package versus usual care to increase uptake of screening for diabetic retinopathy	High patient retention and adherence
Mwangi et al., 2020 [50]	Kenya Rural	N = 104; Intervention arm: 51 participants + 2 peer educators; Control arm: 53 participants + 2 peer educators	Cluster randomized controlled trial	Intervention group: Peer educators delivered monthly DSG-based eye-health education and individual telephone reminders to attend screening versus usual monthly meetings without eye health education	Evidence of feasibility and acceptability of intervention
Park et al., 2015 [51]	Kenya Urban and rural	148 adults above 18 years	Pre-post implementation study	A 6-month diabetes self-management support (DSMS) program–peer leaders guided bimonthly group meetings on self-empowerment, and problem solving	Clinical outcomes: Improvement in HbA1c and systolic blood pressure Acceptability– 9 of 12 groups elected to continue meetings after end of study and stipend support
Pastakia et al., 2015 [52]	Kenya Urban and rural	137 adult patients at a referral and district hospital enrolled for 6 months in SMBG program	Retrospective, observational cohort study	SMBG education provided by peer educators; telephone-based support	Clinical outcomes: significant reduction in median A1C after 6 months of participation; reduction in blood glucose levels
Pastakia et al., 2016 [53]	Kenya Rural	879 adults screened for hypertension and diabetes	Comparison groups	Pilot BIGPIC intervention–with group based education	Acceptability: 72% of screen positive participants returned for subsequent care; 70% remained in care for 9 months of group care Clinical outcomes: significant drop in BP after 9 months of group care Linkages: attracted additional patients; access to capital and financial liquidity in the microfinance component
Tusubira et al., 2021 [57]	Uganda Rural	19 adult patients from outpatient NCD clinics at 3 health facilities	Qualitative design	Exploring self-care practices for hypertension and/or diabetes	Preference for conventional medicine but use of traditional medicine networks of family and peers provided instrumental and emotional support
Leung et al., 2020 [54]	Kenya Rural	31 participants for the pilot study group	Qualitative design using mabaraza and FGDs	Pilot BIGPIC model consisting of microfinance and monthly medical care visits with CHWs	Design of a model of NCD delivery consisting of microfinance and group medical visits; medical availability, financial resources, peer support, and reduced caregiver burden
Thuita et al., 2020 [55]	Kenya Urban and rural	153 adults with diabetes in PHC setting (Intervention_1_ = 51, Intervention_2_ = 51 and control = 51)	RCT with 2 intervention groups and one control group	A nutrition education program with peer-to-peer support (NEP), nutrition education program only (NE), and standard care. Education program conducted 2 h per week for 8 weeks and weekly peer-to-peer interactions for 8 weeks for the NEP group. Follow-up sessions for 6 months for all groups.	Metabolic syndrome (MetS) improved in the NEP and NE groups but worsened in standard care group Improvements in mean values of blood lipids, fasting blood glucose and HbA1c in all the groups with NEP showing greatest improvements followed by NE
Ruchman et al., 2021 [58]	Kenya Rural	2890 patients above 35 years with diabetes or hypertension; 2020 were women	Cross-sectional: analysis of baseline data from participants enrolled in the BIGPIC study	N/A	Participants with trust network alters reported good diet and physical activity; an inverse relationship between advice-network SNCs and elevated SBP
Venables et al., 2016 [56]	Kenya Urban	106 patients with HIV and/or NCD +health care workers	Qualitative: 10 FGD, 19 IDI, 15 sessions of participant observation	N/A	Acceptability of MACs because: time saving, prevented unnecessary queues in clinic, provided health education and group support

## Results

**Fig 1:**
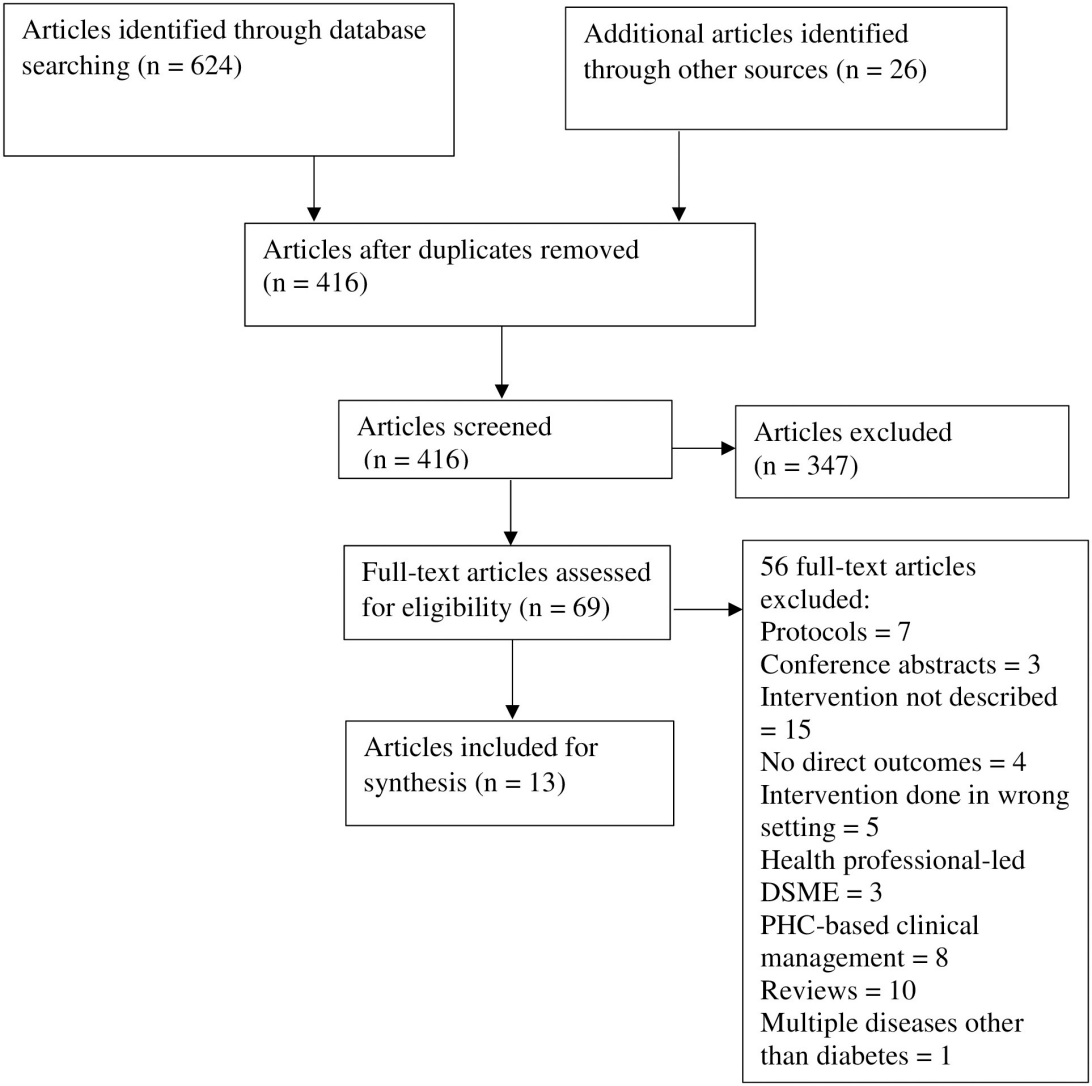
Literature search flow diagram.

The search identified 624 articles. 26 articles were added from hand searches. 234 duplicate articles were removed, yielding 416 articles for title and abstract screening (Fig 1). After screening the titles and abstracts for relevance, 347 articles were excluded. 72 full-text articles underwent full-text review from which 13 articles that met inclusion and exclusion criteria were selected for analysis. Studies were excluded at full-text assessment because they were reviews (N = 10), did not describe a peer support or social networking intervention (N = 15), did not describe a direct outcome of an intervention (N = 4), protocols (N = 7), conference presentations (N = 3), were interventions conducted in multiple countries including Kenya or Uganda (N = 5), described exclusively primary health care-based management interventions (N = 8), described interventions for multiple diseases (N = 1) and were exclusively health professional-led diabetes self-management interventions (N = 3). The study selection process is presented in Fig 1.

### Characteristics of the identified studies

A total of 13 studies were reviewed. Majority of the studies were conducted in Kenya (*n* = 11) and two in Uganda. Their designs were as follows: randomized controlled trials or studies with a pre-post design (n = 4), retrospective comparisons and cohort (n = 4), mixed methods (*n* = 1), cross-sectional (*n* = 1) and qualitative (*n* = 3). Eleven studies [46–56] focused on peer support while two [57, 58] demonstrated the role of social networks in diabetes management. The studies were conducted between 2013 and 2021. The total sample size for the 13 studies was 7,317. Information on included studies is described in Table 1.

### Intervention characteristics

The interventions varied in length from 3 months to 12 months. Patients receiving “routine care” or “usual care” were designated as control groups. Care services were provided as follows: diabetes clinics in PHC facilities (*n* = 8), community settings (*n* = 5); and distributed in urban (*n* = 2), rural (*n* = 7) and peri-urban and rural (*n* = 4) settings.

The criteria for selection of participants for interventions ranged from recruitment from clinics during routine follow-up or after sensitization and/or satisfaction of specific diabetes-related control criteria [47, 48, 51–53, 55] entire diabetes support groups [49, 50] and by use of multiple approaches [46, 54, 56].

Interventions were delivered by nurses [47], peer supporters/educators [49, 50, 55, 56], peer leaders [51], community health workers [48] and multidisciplinary teams [46, 52–54]. Training for non-healthcare professionals including peer supporters, peer educators and community health workers (CHW) to deliver the interventions ranged from one day [46, 48] to 4 weeks [51]. The roles of peer educators/diabetes supporters and/or CHWs were delivering self-management education and support including nutrition and health education [49, 50, 55, 56], telephone-based support and referral [46, 48, 49, 50, 53, 55] and facilitating group meetings and liaison [51, 53, 54].

Interventions involved Medication Adherence Clubs (MAC) [47, 56], microfinance and group medical visits [53, 54] and DSME and other interventions [46, 48–52, 55]. DSME interventions focused on behavioral assessment, goal-setting, problem solving and social support [51, 52, 55, 56] and were supported telephone reminders [46, 48–50, 53, 55]. Some interventions required the participation of multiple stakeholders. For instance, in one intervention [53], CHWs worked through village elders and chiefs and used physical meetings and mobile phone to guide the community on forming self-selected peer microfinance groups of between 10–30 members.

### Outcomes of interventions

#### Learning outcomes

Two studies assessed diabetes knowledge of patients as an indicator of learning outcomes. One study [51] reported no significant changes in diabetes knowledge after 6 months of intervention. Another study [56] measured knowledge in terms of knowledge of other diseases gained by members in integrated MACs. A third study [50] assessed learning in terms of confidence and recognition of peer supporters after training and task shifting.

#### Behavioral outcomes

Diabetes-related behavioral outcomes reported were medication adherence and compliance with protocols, eating behaviors, physical activity and acceptability and loss to follow up (LTFU).

Medication adherence and compliance with protocols were reported in a single study [47] which reported high compliance with protocols including blood pressure and weight checked and blood tests ordered.

Two studies reported on physical activity. One [58] reported sufficient levels of physical activity for participants with any trust network alters. The number of advice and multiplex network alters also had positive associations with sufficient physical activity. Another study [55] found significant changes in physical activity at six months post-intervention.

Eating behaviours were reported in three studies. Two [46, 55] reported significant changes in dietary patterns post-intervention while one [58] found positive associations between social network characteristics (SNCs) representing social cohesion e.g., trust networks and better diet.

Acceptability was reported in 10 studies; two [47, 48] reported low acceptability of intervention measured as overall low referral and low rate of follow up for patients who met the threshold respectively. Eight [46, 49–54, 58] demonstrated high acceptability of interventions. In five studies [49, 50, 53, 54, 56] the reasons given for high acceptability of interventions were: involvement of all stakeholders in implementation, use of the referral card to navigate interactions with health care providers, earning incentives through self-management and financial payment of accumulated savings and interest, and savings in time, money and reduced clinic visits.

Retention rate/loss to follow up was reported in eight studies [46–53]. Only one [48] reported low retention rate due to unwillingness to get to the referral hospital.

#### Clinical outcomes

Clinical outcome indicators assessed included: glycated hemoglobin (HbA1c), body mass index (BMI)/weight, systolic/diastolic blood pressure (SBP/DBP), blood sugar/glucose and hip circumference (HC) and waist circumference (WC). HbA1c was measured in four studies [46, 51, 52, 55] which reported significant improvements in patients’ HbA1c levels.

BMI was reported in three studies; two [51, 58] found no significant changes in BMI and one [55] reported a significant reduction in weight and BMI in the intervention group 6 months post intervention.

Blood pressure was assessed in five studies; four [46, 51, 53, 55] reported statistically significant reductions in blood pressure, and one [58] reported contradictory findings. The number of advice network alters and mean number of activities shared with them negatively related with SBP while the same social network characteristics (SNCs) in the multiplex network positively related with SBP.

Blood glucose or sugar was an outcome measure of three studies. Two studies [52, 53] reported statistically significant reduction in blood glucose, while one [55] found no significant group differences after 6 months post intervention.

Other clinical outcomes including lipid profiles, cholesterol and high-density lipoprotein and overall metabolic syndrome (MetS), were the outcome measures of one study [55] which reported significant reduction in waist and hip circumference in the intervention groups; significant increase in high density lipoprotein (HDL) and significant differences in total cholesterol (TC) levels; reduced metabolic syndrome (MetS) in intervention as compared to control group; significant reduction in prevalence of participants having increased waist circumference, elevated blood pressure, increased serum triglycerides (TG) and reduced HDL among other indicators in the intervention groups. Overall, compared to control group, intervention groups showed a significant increase in participants having less than three MetS factors.

#### Other outcomes

Social support was reported by four studies. One [56] reported that membership in MACs enabled social support through the group environment and integration that reduced stigma. Two studies [46, 57] reported informational support (receiving helpful advice and encouragement to contact the clinic and information about self-care practices), instrumental and emotional support (talking to someone else about diabetes) from other group members. One study [54] reported improved access to medical services by mitigating the need to travel, decreased medication cost, provided peer support and medical reliability. Another study [57] also reported that patients relied on social support from family members and patient peers to mitigate the impact of uncertain access to prescribed medicines. Children provided emotional and instrumental support (money for medicines, transport and household necessities) to sustain self-care. Wives provided husbands emotional support and diet needs while dependent on husbands for financial support.

### Efficacy of interventions

The efficacy of reviewed interventions was assessed using established benchmarks [34] where peer support serves four main functions: assistance in daily management, social and emotional support, linkage to clinical care and ongoing availability of support.

#### Assistance in daily management

Some studies reviewed demonstrate evidence of more practical aspects of diabetes self-management. For instance, patient groups supported each other in acquiring medicines, food and transport [57], and part of the training curriculum and/or diabetes education in some interventions [46] emphasized areas of diabetes self-care including healthy eating, being active and problem solving for better quality of life. Setting and sharing weekly goals on changes in eating behavior and physical activity behavior was also emphasized [55].

Face-to-face meetings were complemented by telephone support [46, 48–50, 52, 53, 55] to encourage daily disease management, drug adherence, self-monitoring of blood glucose, reminders for appointments, and informational support in diabetes care.

Peer supporters also provided specific services that encouraged daily self-management. In one study [50] peer supporters accompanied participants to the eye clinic and waited with them. Interventions involving microfinance and group medical visits [53] demonstrate that receiving loans through Village Savings and Loan Association (VSLA), or savings and interest over 12 months and earning incentives through self-management guarantees access to finances which improves the ability to pay for healthcare.

An intervention using a reciprocal model [46] ensured that participants benefited by providing as well as receiving support. Both champions and partners were able to initiate contacts and provide supportive communication. Consequently, this increased perceptions of the quality of care.

#### Social and emotional support

Belonging to groups provided opportunities for social support and resolution of emotional issues. In one intervention [46] peer supporters received training in using supportive communication skills such as active listening. Another study [57] demonstrates the importance of unofficial groups in social and emotional support through watching out for one another and creating expectations for self-care, food relief and meeting appointments. Finally, belonging to MACs provided social support by creating a sense of belonging and social comparison among club members [56].

#### Linkage to clinical care

Reviewed interventions connected peer supporters with healthcare professionals as well as patients to clinical care beginning from screening. First, being based in diabetes clinics in primary healthcare facilities, or in local diabetes support groups increased linkage to clinical care and increased retention rates. For instance, one study [46] reported increased contact with the diabetes clinic nurse, either through telephone or attending in-person. Second, CHWs acted as a bridge in interactions between patients and professional health care staff [48, 53]. Third, in some interventions, the provision of a referral card opened avenues to clinical care and appointment tracking. For instance, participants in one intervention [50] perceived the referral card as having made it easier to navigate interactions with eye care providers. In four interventions assessed [46, 50, 52, 53], providing a mobile phone or linking participants to a prepaid network improved disease management. Fourth, professional healthcare providers participated in interventions e.g., accompanied CHWs to households to confirm the correct techniques used in counselling, and measuring blood sugar and blood pressure [48]; and joined CHWs in monthly group medical visits [54]. This increased the confidence of peer supporters and the credibility of the intervention [50]. Finally, comprehensive microfinance-linked interventions for instance, the BIGPIC model [53] increased by three times the odds of linkage to care compared to the traditional facility-based care model. Overall, interventions demonstrated higher linkage frequencies to care.

#### Ongoing availability of support

Certain features of reviewed interventions show evidence of feasibility and sustainability. In one intervention [53], group members made decisions on meeting places and convenient time. In two studies [46, 51], interventions continued after the end of the program indicating the long-term attractiveness of the intervention to participants. For instance, in one study [51], 9 out of 12 groups continued peer group meetings after the intervention ended. In another study [46], “buddies” were scheduled for appointments on the same day to facilitate ongoing support 18 months after the intervention.

Interventions focusing on microfinance emphasize community-based recruitment approaches and leverage on CHWs who are part of the healthcare system on a long-term basis using them as group liaisons [53, 54]. Further, synergizing the benefits of microfinance with peer support available through group medical visits ensures the continued management of NCDs (specifically hypertension and diabetes). The addition of mobile telephony as an aspect of peer support makes the interventions convenient. Evidence for continued availability of support is found in high retention rates across many interventions assessed. This can be explained by the involvement of multiple stakeholders in planning and execution of interventions.

In primary healthcare that is grappling with huge patient numbers requiring follow up, majority of whom are stable, MACs provide an opportunity to offload single provider clinic visits by coping with appointments that would ordinarily be included in the regular clinic. MACs therefore reduce the burden of regular follow up among stable patients hence allowing clinicians to deal with other pressing cases, save time, provide support and increase patient satisfaction and flexibility of care delivery [47, 56].

#### Challenges in efficacy of interventions

The efficacy of assessed interventions is hampered by recruitment processes. In two studies assessing MACs [47, 56], the process of enrolment into MACs, as in a number of other interventions assessed, was clinician- as opposed to patient-driven. The lack of members’ input in conceptualization reduces the feasibility and acceptability of the intervention. Save for some interventions [49, 50, 54] whose recruitment processes were non-healthcare institution based, other interventions were conceptualized by health professionals. A third type of loosely organized patient groups [57] developed spontaneously in clinic waiting rooms and were not affiliated to any health facilities. These differences in recruitment processes can explain differences in acceptance, follow up and attrition/retention rates. For instance, loss to follow up fluctuated from zero [49] to 78% [48].

Interventions assessed reported that the lack of meeting places resulted in group members holding meetings in clinics which also serve other healthcare needs. On their part, MACs are constrained by clinic settings and opening hours in line with legal limitations of drug dispensing by non-medical professionals [47]. Furthermore, relationships created in MACs do not extend beyond the clinic hence members do not enjoy social support outside the sessions [47]. The lack of discussions of common problems in and outside sessions, visits and staying in touch and other forms of social support makes it difficult for MAC members to accurately conceptualize peer support.

Reviewed studies reported that the efficacy of interventions is hampered by lack of homogeneity in methods of delivery, duration, frequency and content of interventions and training of peer supporters. For instance, the duration of interventions varied from 3 months to 12 months and training of peer supporters varied from one day to 4 weeks, with or without a trainers’ manual. Supervision of peer educators to assess the extent of fidelity to objectives was not consistent across interventions yet contributed to success/failure. In one study [50], two diabetes support groups whose peer supporters made additional calls to the principal investigator had the highest levels of implementation fidelity.

One major impediment to NCD management is poverty as it limits the ability of social networks. As an emerging innovation, the microfinance model reviewed [53, 54] seeks to alleviate the household burden of care and hence address local complex social determinants of health inequity through changes in social network characteristics. Such models leverage on increased phone network in the region for virtual peer support, referral cards and government health insurance extending its benefits to include NCD care. By promoting income generation, microfinance interventions aim to deal with poverty.

The twin challenges of low literacy and poor eyesight hamper the dissemination of DSME. Some interventions reviewed managed the challenge by reading educational materials translated into local languages loudly in group meetings [46].

## Discussion

We used a scoping review to identify 13 studies published between 2000 and 2021 addressing the use of peer support and social networking in the management of diabetes in Kenya and Uganda. Our findings show the existence of different forms and contexts of group-based peer support models. This supports existing literature on the increased use of cost-effective group-based models in sub-Saharan Africa in provision of DSME [24, 28, 59–61].

In line with literature from SSA, peer supporters were trained for short durations, often ranging from one day to four weeks [25, 28, 29]. Identified studies reported mixed learning and behavioral outcomes, but majority reported improvements in clinical outcomes including HbA1c and blood pressure. In evaluating efficacy of interventions using established criteria [34], findings provide support for the four functions of peer support. However, the diversity in study designs, contexts and type of interventions implemented should be considered in interpreting findings. Put together, these findings support previous literature in sub-Saharan Africa on clinical outcomes of peer interventions [25, 28, 62, 63].

Though very few studies focused on social networks, available evidence points to the important role of SNCs including family and friends and other linkages to social capital in daily management of diabetes which supports existing literature [7, 9, 32, 64–66]. These findings on the role of social networks provide a theoretical basis to existing “buddy” models in SSA [26, 46]. However, a contradictory finding on the relationship between SNCs and health outcomes [57] calls for more research.

The review also revealed challenges that hamper the efficacy of interventions including recruitment processes, duration and frequency of intervention, training of peer supporters, poverty, limited literacy and unavailability of meeting places among others. These factors were found to be responsible for differences in retention rates and effectiveness of interventions. Interventions that employed interactive peer-to-peer communication [46, 49, 50] reported higher success rates. Latter models incorporating microfinance-based interventions support the need to deal with socio-economic barriers to healthcare access including financial hardships and cutbacks in financing of healthcare in LMICs as reported in the literature [30, 32, 33, 67, 68]. Additionally, a combination of face-to-face meetings and telephone support appeared to help alleviate problems associated with distance, cut costs and enhance support. This was made possible by providing mobile phones or linking participants to a prepaid network. The findings support prior research in SSA on the partial efficacy of telephone support when used within other interventions [26, 27, 40].

In MACs, the flexibility in scheduling allowed judicious use of time for people living in urban areas who must juggle between earning economic livelihoods and seeking health care. This is especially important since literature [69] suggests that urban residence predicts poor adherence to self-monitoring of blood glucose. Therefore, these findings suggest that MACs can be scaled up for diabetes care as they are for HIV [70] and hypertension care [71] in sub-Saharan Africa. However, one major challenge identified among MACs include anonymity which limited the ability to forge strong bonds. This was associated with the clinician-related nature of recruitment and the lack of durable interactions among club members.

Finally, some interventions demonstrated evidence of sustainability by involving diverse stakeholders in conceptualization and execution; and by working within existing healthcare systems and networks, including community health workers, diabetes support groups, administrators and chronic disease networks. By leveraging on existing networks, such interventions cut costs, reduce stigma for NCDs and gain from synergy.

### Limitations

First, our search was limited to articles published in English and indexed in three databases–PubMed, ScienceDirect and Cochrane Library. We may have missed important articles written in other languages and in other databases or other informal sources not reviewed, or not publicly available. Second, included studies employed diverse designs which were not subjected to methodological quality assessment. Third, the review is based on 13 studies majorly from Kenya which may limit the strength of conclusions derived. Fourth, the inability to determine which kinds of interventions were found to be effective draws from the broad aim of this scoping review to rapidly map evidence supporting peer support and social networking interventions.

## Conclusions

The limited number of studies provide insufficient evidence to make concrete conclusions on the efficacy of interventions. However, findings shed light on the formats and strategies used, and provide some evidence for the acceptability, feasibility and scalability of peer support and social networking in diabetes care. Although further research should identify the specific activities, processes and interventions that were highly successful, these findings provide a basis for debate among stakeholders in NCD care. As emerging evidence suggests, peer support and social networking may be promising approaches to NCD self-management.

## Supporting information

S1 Checklist PRISMA extension for Scoping Reviews (PRISMA-ScR) checklist. (DOCX)

S1 Appendix Sample search strategy (PubMed). (PDF)
